# Development of siRNAs targeting interferon beta as a novel therapeutic approach for adult and juvenile dermatomyositis

**DOI:** 10.3389/fimmu.2026.1851483

**Published:** 2026-10-01

**Authors:** Joanna E. Parkes, Andrés F. Correa-Sánchez, Inês Fial, Isaac Tam, Kaladevi Selvam, Mark Cunningham, Nick Lench, Peter L. Oliver

**Affiliations:** Medical Research Council Nucleic Acid Therapy Accelerator (NATA), Research Complex at Harwell, Didcot, United Kingdom

**Keywords:** autoinflammatory, dermatomyositis, interferon beta, nucleic acid therapy, siRNA

## Abstract

**Background:**

A key feature of the autoimmune conditions adult and juvenile dermatomyositis (DM/JDM) is the correlation of interferon (IFN) beta induction with disease initiation and activity. Our aim was to establish a short interfering RNA (siRNA) approach to knockdown *IFNB1* expression as a therapeutic strategy for DM/JDM.

**Methods:**

A library of 74 siRNAs targeting *IFNB1* was designed and screened for *IFNB1* knockdown by qRT-PCR. Lead siRNAs were analysed for secreted IFN beta protein reduction and acute cytotoxicity. IFNB1 expression was induced for efficacy screening *in vitro*, including in immortalised myoblasts, myotubes and dermal fibroblasts, alongside JDM patient-derived myotubes. RNA sequencing was also performed on siRNA treated and untreated dermal fibroblasts and patient myotubes.

**Results:**

Initial screening of 27 siRNAs identified two lead siRNAs with significant knockdown of both IFN beta transcript and secreted protein in all cell lines, with one lead siRNA demonstrating a more favourable cytotoxicity profile. RNA sequencing and pathway analysis revealed that both lead siRNAs were able to reduce the induction of disease-associated immune and autoimmune pathways in dermal fibroblasts and JDM myotubes. An additional 47 siRNAs across the *IFNB1* locus were screened; however, the previous lead siRNA remained the most active across relevant cell lines.

**Conclusions:**

Overall, comprehensive *in vitro* screening identified a lead siRNA targeting *IFNB1* with significant mRNA and protein knockdown activity, low cytotoxicity and the ability to modulate immune-relevant pathways in muscle and skin cells. This is a promising first step towards the development of an siRNA therapy for DM/JDM and other conditions driven by IFN beta over-expression.

## 1 Introduction

Myositis refers to a heterogenous group of rare autoimmune conditions characterised by inflammation and weakness of skeletal muscle but also featuring extra-muscular manifestations in the skin, lungs, heart, gastrointestinal tract and joints (1). The myositis subtypes adult and juvenile dermatomyositis (DM/JDM) affect skin in the form of rashes and papules which may even occur without detectable muscle involvement (2). DM/JDM is currently treated with immunosuppressive drugs and glucocorticoids which are not effective in all patients and have significant side effects (3, 4). Therefore, novel treatments against disease-specific therapeutic targets are needed for these debilitating conditions. A type I interferon (IFN) signature is observed across the subtypes of myositis patients but is generally more striking in DM/JDM and correlates with disease activity in both DM and JDM (5–12). The IFN signature in DM skin is highly correlated with expression of the IFN beta transcript, *IFNB1* (13), and circulating IFN beta protein levels correlate with cutaneous disease activity in DM patients (14). Furthermore, *in vitro* studies have shown that treatment of healthy cells with IFN beta can induce cellular phenotypes associated with DM/JDM, such as proangiogenic properties and mitochondrial dysfunction (15, 16). Notably, there is a case study of a patient receiving IFN beta treatment for multiple sclerosis who was subsequently diagnosed with DM; this was exacerbated by further IFN beta injections (17). Overall, there is convincing evidence that IFN beta is both correlated with disease and has a causative role in initiating or maintaining the disease phenotype in DM/JDM.

We propose targeting IFN beta at the transcript level using a nucleic acid therapy as a novel therapeutic approach for DM/JDM and other conditions with pathological overexpression of IFN beta. IFN beta is particularly amenable for a nucleic acid therapy approach using short interfering RNAs (siRNAs) for gene-specific knockdown, since there is a single *IFNB1* transcript consisting of a single exon (18); therefore, there is no concern that tissue- or cell-specific isoforms of the gene will be missed, and siRNAs can be generated to avoid any possible cross-reactivity with other *IFN* genes.

Here, we designed, screened and further characterised a panel of siRNAs targeting the IFN beta transcript in multiple disease-relevant cell lines as a first step toward developing an siRNA therapy for DM/JDM and other autoinflammatory conditions.

## 2 Materials and methods

### 2.1 siRNA design

Oligonucleotides were designed targeting the *IFNB1* mRNA transcript NM_002176.4. A library of 21-nucleotide (nt) sequences was generated across the entire transcript. Exclusion criteria for sequences included: lower than 40% or greater than 60% GC content, single-nucleotide stretches of four or more, predicted secondary structures such as hairpin loops, and potential sequence-driven off-target interactions assessed by BLAST searches against the pre-spliced human transcriptome. The selected siRNA scaffold consists of a modified Advanced Enhanced Stabilisation Chemistry (Adv. ESC) pattern using 2′-O-Methyl (2′-OMe) and 2′-fluoro (2′-F) modified nucleotides (**Supplementary Figure S1B**) (19). Additionally, a two nucleotide dT overhang at the 3 ′ end of the antisense strand and two phosphorothioate bonds at both ends of the duplex were introduced to increase stability against exonucleases (20, 21).

### 2.2 Oligonucleotide synthesis

Oligonucleotides were synthesised by solid-phase synthesis on a MerMade192X synthesiser (LGC Biosearch Technologies) using 200 nmol-scale pre-packed columns of 1000 Å Universal Controlled Pore Glass (CPG) (loading 47.7 μmol/g, LGC Biosearch Technologies). Cleavage of the 4,4′-dimethoxytrityl group was performed with 3% trichloroacetic acid in dichloromethane (LGC Biosearch Technologies). 2′-O-methyl phosphoramidites, 2′-fluoro phosphoramidites, and dT phosphoramidites were prepared as 0.1M solutions in dry acetonitrile (LGC Biosearch Technologies). 0.25 M 5-ethylthio-1H-tetrazole in dry acetonitrile (LGC Biosearch Technologies) was used as an activator. 0.1 M 3-ethoxy-1,2,4-dithiazoline-5-one (EDITH) in acetonitrile (LGC Biosearch Technologies) was used as sulphurisation reagent. 0.02 M iodine in tetrahydrofuran/pyridine/water (7:2:1, v/v/v, LGC Biosearch Technologies) was used for the oxidising steps. A 1:1 mixture of Capping Mix A (tetrahydrofuran/lutidine/acetic anhydride, 8:1:1, v/v/v, LGC Biosearch Technologies) and Capping Mix B (16% N-methylimidazole in tetrahydrofuran, LGC Biosearch Technologies) was used for the capping steps. Cleavage and deprotection of the oligonucleotides were performed using 33% aqueous ammonia for 8 h at 55 °C.

Purification of the oligonucleotides was performed on the Waters ACQUITY AutoPrep HPLC system (XBridge BEH C18 OBD Prep column; 130 Å; 10 × 250 mm, 5 μm; Waters) at 65 °C with a flow rate of 0.8 ml/min. Mobile phase A: 0.1 M triethylammonium acetate in water; Mobile phase B: 0.1 M triethylammonium acetate in 50% acetonitrile. The liquid chromatography was monitored by UV at 260 nm.

The identity of oligonucleotides was verified by liquid chromatography mass spectrometry (LC-MS) analysis on a Waters SYNAPT XS Mass Spectrometer (ACQUITY PREMIER Oligonucleotide BEH C18 column; 130 Å; 2.1 × 50 mm, 1.7 μm; Waters) at 65 °C with a flow rate of 0.3 ml/min. Mobile phase A: 7 mmol/l triethylamine (TEA), 80 mM hexafluoroisopropanol (HFIP) in water; Mobile phase B: 3.5 mmol/L TEA, 40 mmol/l HFIP in 50% ACN; gradient 5% to 30% B in 8 minutes. The purity of the oligonucleotides was assessed using the integrated chromatography peaks monitored by UV (λ =260 nm). Only oligonucleotides with purity ≥ 90% were considered for *in vitro* assays. MS spectra were taken in negative mode using electrospray ionisation. Samples were filtered on a Ultrafree-MC Centrifugal Filter (0.22 μm, 5 minutes, 14,000 rpm, Millipore) and quantified in triplicate by measuring the absorbance at 260 nm (NanoDrop One, Thermo Scientific). Duplex formation was achieved by annealing equimolar amounts of sense and antisense strands in water and heating at 95 °C for 5 minutes and then allowed to cool gradually to room temperature.

### 2.3 Cell culture

Immortalised human myoblasts (KM1421) derived from paravertebral muscle of a female child were obtained from the platform for immortalization of human cells from the Institut de Myologie, Paris. Primary myoblasts derived from muscle tissue of a healthy adult male and from a male child with JDM were from The Biobank of Cells, tissues and DNA from patients with neuromuscular diseases, member of the Telethon Network of Genetic Biobanks (project no. GTB12001), funded by Telethon Italy, and of the EuroBioBank network.

Myoblasts were cultured in flasks coated with 0.4% porcine gelatin (Sigma-Aldrich) using skeletal muscle growth medium (Promocell) with 20% foetal bovine serum (FBS) and 1% penicillin/streptomycin. Differentiation into myotubes was induced by switching to Dulbecco’s modified Eagle’s medium (DMEM) with 2% horse serum and 1% penicillin/streptomycin for 5–10 days. Immortalised human dermal fibroblasts (Innoprot) were cultured in flasks coated with 0.4% porcine gelatin using Fibroblast Media 2 (Sciencell or Promocell) with 5% or 2% FBS (according to manufacturer) and 1% penicillin/streptomycin. Prior to transfection, media was replaced with low serum media (DMEM with 0.1% FBS, 1% penicillin/streptomycin). A549 human lung carcinoma cells (ECACC, Sigma-Aldrich) were cultured in DMEM with 10% FBS and 1% penicillin/streptomycin.

### 2.4 *IFNB1* induction and siRNA transfection

*IFNB1* expression was induced by stimulating toll-like receptor 3 (TLR3) with a synthetic analogue of double stranded RNA, polyinosinic–polycytidylic acid sodium salt (Poly I:C) (Sigma-Aldrich). Optimisation experiments were conducted to establish the concentration of Poly I:C and volume of Lipofectamine 2000 (Invitrogen) complexed in Opti-MEM reduced serum media (Gibco) used for transfection for each cell line. After 24-hours, cells were transfected with siRNAs complexed with Lipofectamine RNAiMAX (Invitrogen) in Opti-MEM for 48-hours. Transfection was performed in triplicate in 24-well plates for muscle and skin cells and in quadruplicate in 96-well plates for A549 lung cells. Opti-MEM was added to untreated (UT) control wells.

### 2.5 RNA extraction and quantitative reverse transcription polymerase chain reaction

RNA was extracted from cells using the MagMAX miRVana Total RNA Isolation kit on the Kingfisher Flex System (Thermo Fisher Scientific). Reverse transcription was performed using the High Capacity cDNA conversion kit with RNase inhibitor (Fisher Scientific). qRT-PCR was performed in triplicate using TaqMan Fast Advanced MasterMix and TaqMan Gene Expression Assays for *IFNB1* (Hs01077958_s1)*, HPRT1* (Hs02786624_g1) and *GAPDH* (Hs02786624_g1) (Applied Biosystems) and run on the Bio-Rad CFX Opus 384 Real-Time PCR System. Results were analysed using CFX Maestro software v2.3 (Bio-Rad).

### 2.6 Quantification of secreted IFN beta protein

Culture media collected from wells immediately before RNA isolation was centrifuged and supernatant from a subset of wells was used in duplicate in an enzyme-linked immunosorbent assay (ELISA) (Abcam ab278127) to detect secreted IFN beta protein. Values were calculated from 450 nm absorbance readings in duplicate from two wells per group using a Clariostar Plus plate reader (BMG Labtech, software version 5.70 R2). Normalised values were calculated by subtracting the background signal (average absorbance for the blank control) and converted to pg/ml based on an IFN beta standard curve.

### 2.7 Cytotoxicity assays

Apoptosis and necrosis were measured over 72 hours using a RealTime-Glo Annexin V Apoptosis and Necrosis Assay (Promega). Cells were plated in a 96-well opaque white plate and transfected with a range of concentrations of siRNAs as described above and then detection reagent was immediately added. Poly I:C induction was not performed to avoid noise from signal not directly related to toxicity from the siRNA. Luminescence and fluorescence signals were measured at several timepoints from 0–72 hours to determine levels of apoptosis and necrosis, respectively using the following settings on a Clariostar Plus plate reader (BMG Labtech, software version 5.70 R2). Luminescence: top optic with no filters, enhanced dynamic range, measurement interval 1.0s and focal height 11mm; Fluorescence: top optic, excitation 482-16, Dichroic 500; Emission 530-40; 0.2s settling time; 40 flashes; with enhanced dynamic range and auto focus for the first measurement to set the focal height. Normalised values were calculated by subtracting the background signal (average for no-cell control wells).

### 2.8 RNA sequencing and pathway analysis

RNA samples were purified from a total of 27 different treatment conditions in biological triplicates. RNA sequencing was carried out at Lexogen GmbH (Vienna, Austria) using Lexogen CORALL mRNA-Seq library preparation in shared lanes with average read depth of 20.4 million per sample. Data analyses by Lexogen included demultiplexing, alignment to a reference genome using STAR aligner, trimming using cutadapt, and quality control using MultiQC. In summary, all libraries generated data representing 79.19% to 87.44% uniquely mapped reads, with read distribution for the coding exons from 57.46% to 63.31% and introns from 3.73% to 6%. Expression correlation between biological replicates was very high, ranging from R^2^ = 0.932 to 0.956 using orthogonal regression. The summary file of unique counts was used as input for analysis on Integrated Differential Expression & Pathway analysis (iDEP.96 and iDEP v2.3.2) to convert gene IDs, filter for genes with ≥ 0.5 counts per million, transform counts using EdgeR: log2(CPM + c) and perform principal component analysis (22). Heatmaps of hierarchical clustering were generated using Pearson’s correlation coefficient for the distance matrix and average linkage. Differential gene expression was analysed using DESeq2 with false discovery rate (FDR) adjusted p-value <0.01, fold change ≥2 and independent filtering of lower counts for selected comparisons to identify statistically and biologically significantly differentially expressed genes. Pathway analysis was performed using Gene Set Enrichment Analysis (GSEA) with pre-ranked fgsea and pathway significance cutoff of FDR < 0.01 for the top 20 most significantly dysregulated KEGG pathways. Differential expression and pathway enrichment plots were generated using ggplot2 (23).

### 2.9 Statistical analyses

For ELISA assays, cytotoxicity assays and RNA-seq clustering profiling, statistical analysis was performed on normalised values using GraphPad Prism version 10.5.0. with ordinary one-way ANOVA to compare all groups to the selected control/experimental group with Turkey’s multiple comparisons test.

## 3 Results

### 3.1 Screening in immortalised muscle and skin cells identified multiple siRNAs that significantly reduced IFN beta expression

siRNAs targeting *IFNB1* were designed across both untranslated regions (UTRs) and the coding region; following hierarchical filtering against exclusion criteria, a library of 74 siRNAs were selected (**Supplementary Figure S1A**). An initial panel of 27 siRNAs was synthesised, purified and subjected to in-house quality controls before being taken forward for *in vitro* efficacy screening. DM and JDM are characterised by inflammation of skeletal muscle and skin rashes (1); therefore, the panel of siRNAs was screened initially in both skeletal muscle cells (myoblasts and myotubes) and dermal fibroblasts. To model inflammatory *IFNB1* induction in the screening process, cells were first transfected with Poly I:C, a synthetic analogue of double stranded RNA which stimulates TLR3 signalling (24), followed by transfection with a single dose of each siRNA individually.

In immortalised myoblasts, 1 ng/ml Poly I:C treatment induced an 80-fold increase in *IFNB1* expression compared to the untreated myoblast group (MB-PIC versus MB-UT, **Figure 1A**). Importantly, compared to the Poly I:C group, transfection of a non-targeting siRNA control into Poly I:C treated cells did not influence expression of *IFNB1* (MB-PIC versus MB-PIC-NTC, **Figure 1A**). After siRNA library efficacy screening at 30 nM, treatment with seven of the 27 siRNAs resulted in statistically significant knockdown of *IFNB1* expression compared to the Poly I:C group (MB-PIC-4001 to -4030 versus MB-PIC; p < 0.05, **Figure 1A**). Notably, all seven active siRNAs were targeted against the *IFNB1* 5’ UTR (**Supplementary Figure S1A**).

**Figure 1:**
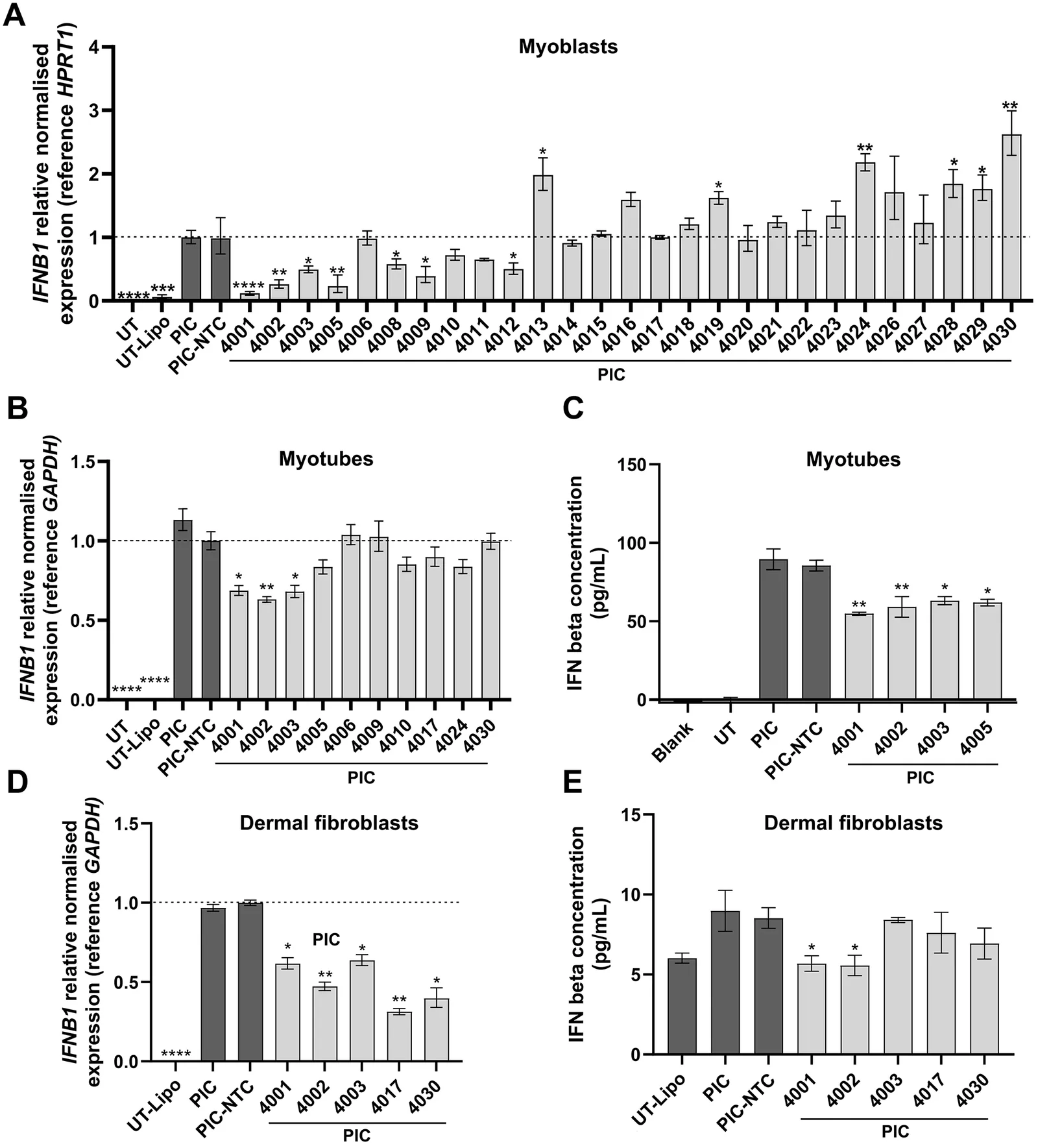
Expression of interferon beta in immortalised muscle and skin cells treated with Poly I:C followed by siRNA targeting *IFNB1.* Immortalised myoblasts were transfected with 1 ng/ml Poly I:C for 24 hours followed by 30 nM siRNA for 48 hours. Normalised relative expression of *IFNB1* measured by qRT-PCR with reference gene *HPRT1* shown relative to the control group non-targeting siRNA (NTC) **(A)**. Myotubes were transfected with 1 ng/ml Poly I:C followed by 20 nM siRNA, dermal fibroblasts were transfected with 10 ng/ml Poly I:C followed by 40 nM siRNA. Normalised relative expression of *IFNB1* with reference gene *GAPDH* and control group non-targeting control siRNA (NTC) for myotubes **(B)** and dermal fibroblasts **(D)**. Concentration of IFN beta in cell culture media supernatant measured by enzyme-linked immunosorbent assay for myotubes **(C)** and dermal fibroblasts **(E)**. Normalised relative expression of *IFNB1*, standard error of the mean and p-values were calculated using Bio-Rad CFX Maestro software. IFN beta concentration values were calculated from 450 nm absorbance readings in duplicate from two wells per group and converted to pg/ml based on an IFN beta standard curve. P-values calculated using ordinary one-way ANOVA comparing to non-targeting control siRNA group. *Indicates p-value <0.05; **<0.01, ***<0.001, ****<0.0001. *UT, non-treated; UT-Lipo, no treatment plus Lipofectamine 2000; PIC, poly I:C treatment; NTC, non-targeting control siRNA; 4001–4030 siRNAs*.

Ten siRNAs with a range of efficacy in myoblasts (the five most active, three inactive and two showing *IFNB1* upregulation) were then screened in differentiated myotubes. Treatment of myotubes with 1 ng/ml Poly I:C induced a 246-fold increase in *IFNB1* mRNA expression compared to the untreated myotube group (MT-PIC versus MT-UT, **Figure 1B**). From the 10 siRNAs tested, treatment with three of the most active siRNAs at 20 nM resulted in a statistically significant knockdown of *IFNB1* expression compared to the corresponding NTC group (MT-PIC-4001 to -4030 versus MT-PIC-NTC; p < 0.05, **Figure 1B**). To next assess IFN beta protein knockdown in treated myotubes, the cell culture supernatants were analysed by ELISA after dosing with the four most active siRNAs (MT-PIC-4001, -4002, -4003, -4005). All four of these siRNAs caused a significant reduction of the detected IFN beta protein compared with the MT-PIC-NTC group, as predicted by the qPCR data (**Figure 1C**).

Three of the most active siRNAs (4001, 4002 and 4003), alongside siRNAs which were inactive or increased expression (4017 and 4030, respectively, **Figures 1A–C**), were then tested in immortalised dermal fibroblasts. Treatment of dermal fibroblasts with 10 ng/ml Poly I:C induced a 361-fold increase in *IFNB1* mRNA expression compared to the Lipofectamine 2000-only group (DF-PIC versus DF-UT-Lipo, **Figure 1D**). Dosing Poly I:C treated dermal fibroblasts with two of the most active siRNAs (4001 and 4002) at 40 nM resulted in significant knockdown of *IFNB1* expression at both the RNA and the secreted protein level compared with the corresponding NTC group (DF-PIC-4001/-4002 versus DF-PIC-NTC; p < 0.05, **Figures 1D, E**). In summary, efficacy screening in immortalised muscle and skin cells identified consistently two lead siRNAs (4001 and 4002).

### 3.2 Transfection with lead siRNA 4002 caused no significant increase in cytotoxicity in immortalised muscle and skin cells

A dose-response experiment was performed to assess acute cytotoxicity following transfection of the lead siRNAs (4001 and 4002), alongside siRNAs with variable activity (4003, 4005, 4017 and 4030) in immortalised myoblasts, differentiated myotubes and dermal fibroblasts without Poly I:C induction. Cells were transfected with 0.1–1000 nM siRNA and nine timepoints from 0–72 hours were assessed for markers of apoptosis and necrosis; results from the 24-hour timepoint are shown in **Figure 2**.

**Figure 2:**
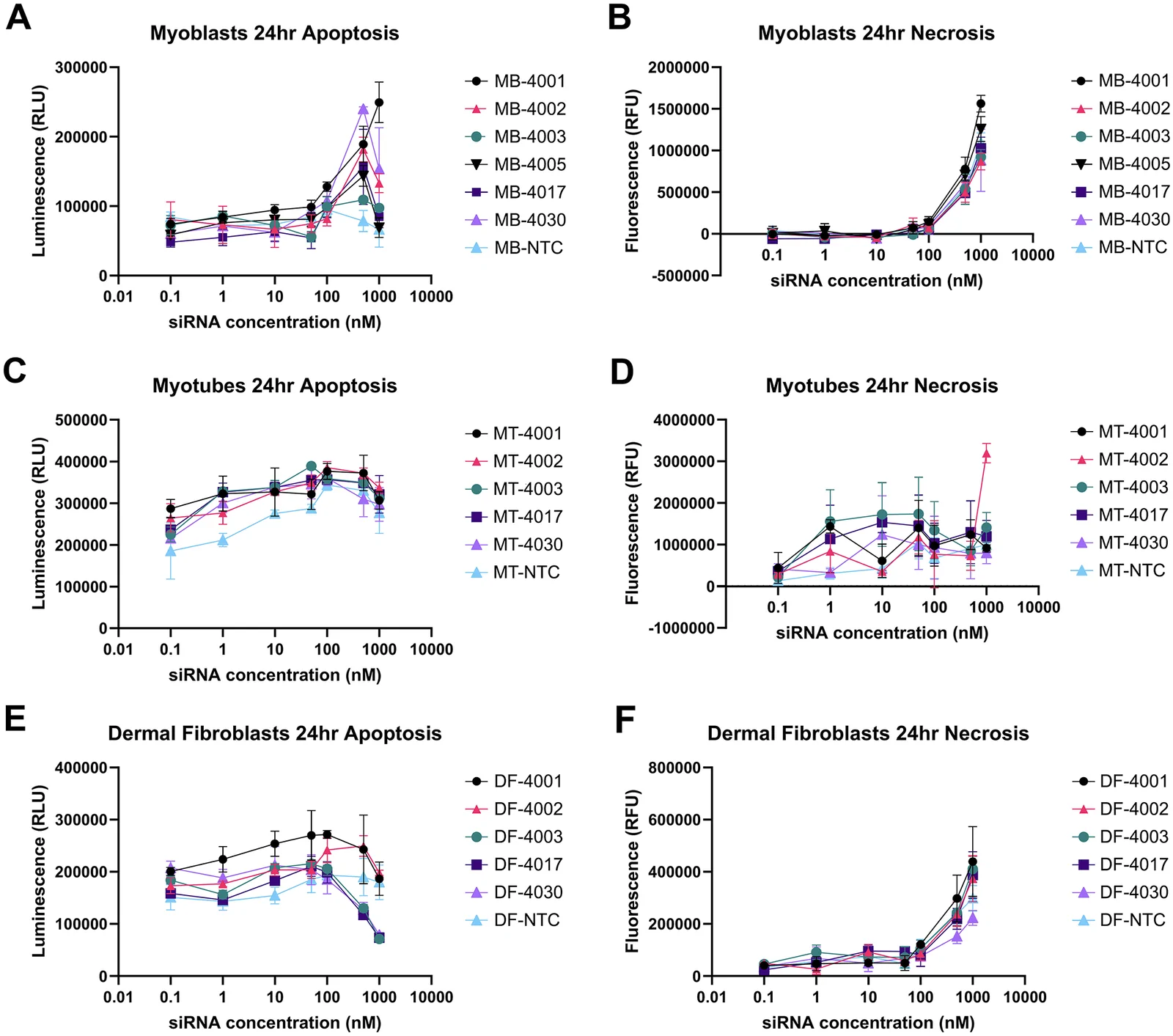
Acute cytotoxicity analysis in immortalised myoblasts, differentiated myotubes and dermal fibroblasts treated for 24 hours with siRNAs targeting *IFNB1*. Cells were transfected with 0.1–1000 nM siRNA (4001–4030) immediately followed by addition of RealTime-Glo Annexin V Apoptosis and Necrosis Assay reagents. Luminescence (apoptosis) and fluorescence (necrosis, excitation 482–16 nm, emission 530–40 nm) signals were measured. Data are shown after 24 hours with three replicates per treatment group for myoblasts **(A, B)**, myotubes **(C, D)** and dermal fibroblasts **(E, F)**. Net luminescence and net fluorescence calculated by subtracting mean reading from six wells without cells or treatment. *NTC, non-targeting control siRNA; 4001–4030 siRNAs.; MB, myoblasts; MT, myotubes; DF, dermal fibroblasts*.

In myoblasts, the apoptosis and necrosis response markers were generally increased at the highest siRNA concentrations. Comparing the lead siRNAs with the NTC, apoptosis response was significantly greater at 500 nM and 1000 nM for both lead siRNAs, but necrosis was only significantly higher for 4001 at 1000 nM (MB-NTC versus MB-4001/-4002; adjusted p < 0.0001, **Figures 2A, B**; **Supplementary Table S1** for all statistical comparisons). In myotubes, the trend was less obvious over the increasing siRNA doses. For apoptosis, the NTC had lower luminescence than all the siRNAs tested at low concentrations; however, there was no significant difference between the lead siRNAs and other siRNAs tested (**Figure 2C**; **Supplementary Table S1**). For necrosis, only treatment with siRNA 4002 at the highest dose (1000 nM) induced a significantly higher fluorescence signal than the NTC (MT-NTC versus MT-4002; adjusted p < 0.0001, **Figure 2D**; **Supplementary Table S1**). In dermal fibroblasts there was a slight increase in apoptosis markers over increasing concentrations up to 100 nM and a more marked increase in necrosis markers at the two highest concentrations (500 nM and 1000 nM, **Figures 2E, F**). Compared with the NTC, siRNA 4001 induced a significantly greater apoptosis response at 1 nM to 100 nM and a larger necrosis response at 1000 nM (DF-NTC versus DF-4001; adjusted p < 0.0001 apoptosis, p < 0.001 necrosis), whereas the apoptosis signal from siRNA 4002 was only significantly higher than NTC at 500 nM (DF-NTC versus DF-4002; adjusted p < 0.01, **Figures 2E, F**; **Supplementary Table S1**).

Overall, siRNA 4002 showed the most favourable tolerability profile of those tested, with only the two highest doses causing any significant increase in apoptosis or necrosis response compared to NTC across all three cell types.

### 3.3 Lead siRNA showed significant on-target activity with no significant increases in cytotoxicity in healthy and JDM myotubes

To analyse the response to the lead siRNAs in a JDM context, healthy control and JDM myoblasts were differentiated into myotubes then treated with Poly I:C, followed by transfection of 40 nM of the lead siRNAs 4001 or 4002 in addition to siRNAs 4003, 4017 and 4030 which had variable activity between cell types in the immortalised cells (**Figure 1**). Treatment with 10 ng/ml Poly I:C induced a 99-fold and 94-fold increase in *IFNB1* mRNA expression in healthy control and JDM myotubes, respectively, compared to the corresponding Lipofectamine 2000-only treatment group (HC-PIC/JDM-PIC versus HC-UT-Lipo/JDM-PIC-Lipo, **Figure 3A**).

**Figure 3:**
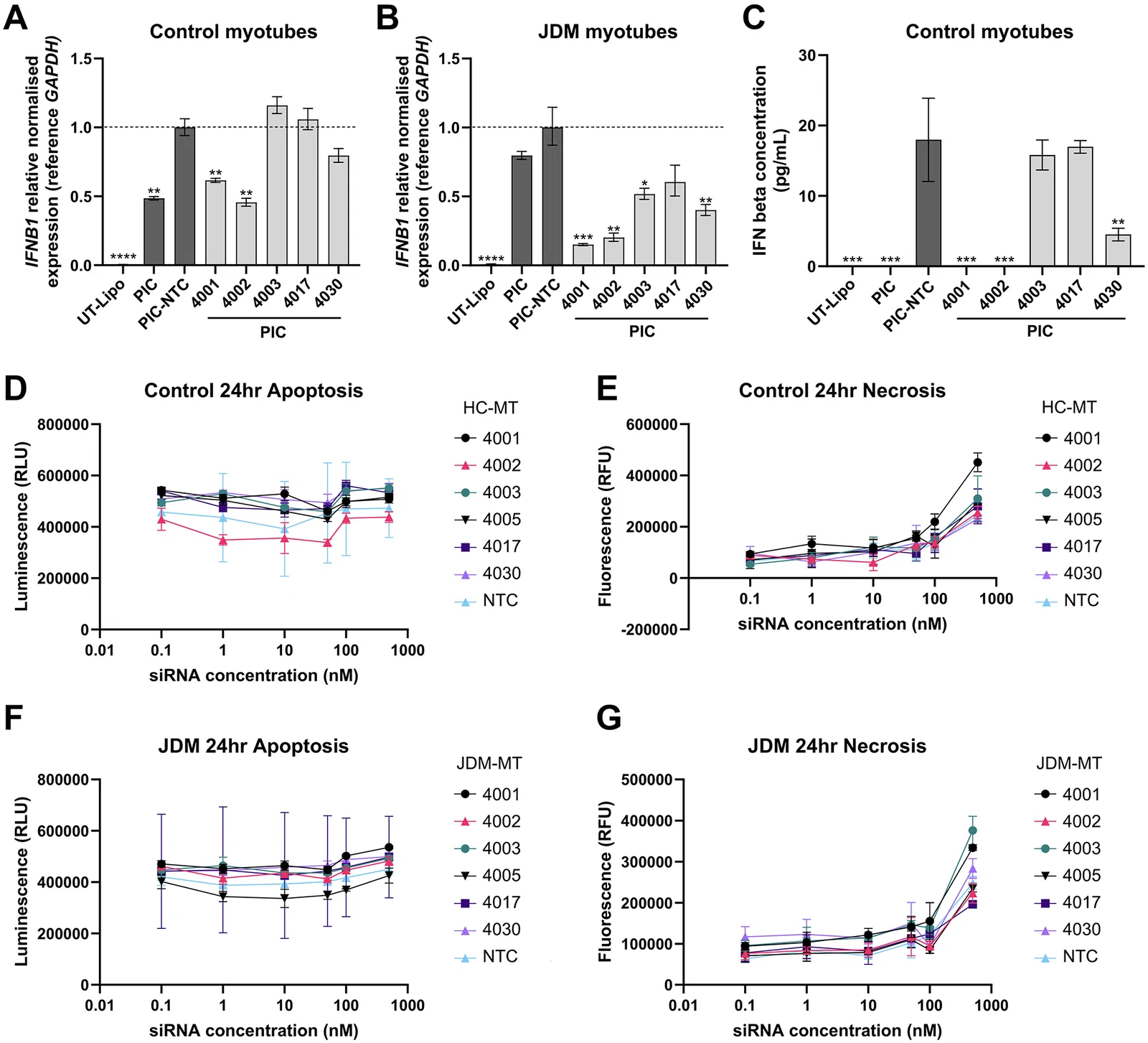
Expression of interferon beta and acute cytotoxicity analysis in healthy control and juvenile dermatomyositis myotubes treated with siRNAs targeting *IFNB1.* For evaluation of IFN beta expression knockdown, differentiated myotubes were transfected with 10 ng/ml Poly I:C for 24 hours followed by 40 nM siRNA for 48 hours. Normalised relative expression of *IFNB1* measured by qRT-PCR with reference gene *GAPDH* and control group non-targeting control (NTC) siRNA for healthy control **(A)** and juvenile dermatomyositis (JDM) **(B)** myotubes. Values were calculated using Bio-Rad CFX Maestro software. Concentration of IFN beta in cell culture media supernatant measured by enzyme-linked immunosorbent assay for healthy control myotubes **(C)**. Values were calculated from 450 nm absorbance readings in duplicate from two wells per group and converted to pg/ml based on an IFN beta standard curve. P-values calculated using ordinary one-way ANOVA comparing to non-targeting control siRNA group. For measurement of acute cytotoxicity, myotubes were transfected with 0.1–500 nM siRNA immediately followed by addition of RealTime-Glo Annexin V Apoptosis and Necrosis Assay reagents. Luminescence (apoptosis) and fluorescence (necrosis, excitation 482–16 nm, emission 530–40 nm). Data are shown after 24 hours with three replicates per treatment group for healthy control **(D, E)** and JDM **(F, G)** myotubes (HC-MT/JDM-MT). Net luminescence and net fluorescence were calculated by subtracting mean reading from six wells without cells or treatment. * Indicates p-value <0.05; **<0.01, ***<0.001, ****<0.0001. *UT-Lipo, untreated plus Lipofectamine 2000; Poly I:C, poly I:C treatment; NTC, non-targeting control siRNA; 4001–4030, siRNAs ; MT, myotubes*.

For healthy control myotubes, treatment with the siRNAs 4001 and 4002 resulted in significant knockdown of *IFNB1* expression compared with the NTC group (HC-PIC versus HC-NTC; p < 0.01, **Figure 3A**). However, the concentration of secreted IFN beta protein in the cell culture media was reduced below the limit of detection for siRNAs 4001 and 4002 (**Figure 3C**). For the JDM myotubes, treatment with siRNAs 4001 and 4002 resulted in the largest degree of knockdown of *IFNB1* expression compared to the NTC group, although dosing with siRNAs 4003 and 4030 also caused significant knockdown (JDM-PIC-4001 to 4030 versus JDM-PIC-NTC; p < 0.001-0.05, **Figure 3B**). The concentration of secreted IFN beta protein in the cell culture media was below the limit of detection of the ELISA for all groups so a comparison could not be made for the JDM myotubes (data not shown). Overall, treatment with siRNAs 4001 and 4002 resulted in significant knockdown of *IFNB1* in both Poly I:C-induced groups, with a larger reduction in expression in JDM myotubes compared to healthy myotubes (80-85% compared to 38-54% knockdown, **Figures 3A, B**).

Cytotoxicity was then assessed as above in the healthy control and JDM myotubes transfected with siRNA doses ranging from 0.1 nM to 500 nM. For all siRNAs tested there was no significant increase in apoptosis with increased treatment concentrations after 24-hours (**Figures 3D, F**) and only an increase in necrosis at the highest dose (500 nM) for both control and JDM myotubes (**Figures 3E, G**; **Supplementary Table S1**). For apoptosis, there was no significant difference between the test siRNAs and the NTC siRNA for either control or JDM myotubes. For necrosis, there was only an increase in signal compared to NTC at the highest dose (500 nM) for siRNA 4001 in control myotubes (HC-NTC versus HC-4001; p ≤ 0.0001, **Figure 3E**) and for siRNAs 4001 and 4003 in JDM myotubes (JDM-NTC versus JDM-4001/-4003; p ≤ 0.0001, **Figure 3G**; **Supplementary Table S1**). Taken together, these data suggest that there is no indication of acute cytotoxicity of these siRNAs at concentrations up to 100nM in patient-derived healthy and JDM myotubes, and that siRNA 4002 has a better tolerability profile than siRNA 4001 at the highest concentrations tested.

### 3.4 Lead siRNAs show specificity for modulation of immune pathways downstream of *IFNB1* knockdown

IFN beta is a key cytokine in the inflammatory response to viral infection. Overexpression of IFN beta in autoimmune diseases including DM/JDM results in tissue damage due to inflammation, inducing further IFN beta signalling in a positive feedback loop of chronic inflammation (25). Our aim was to block this feedback loop and reduce levels of inflammation (and associated tissue damage) by knocking down expression of IFN beta and in turn the activation of downstream inflammatory pathways. Therefore, we applied transcriptomics to gain an understanding of the global impact of *IFNB1* knockdown on downstream pathways and to identify any gene dysregulation that might be caused by off-target toxicity of the siRNAs. Dermal fibroblasts (DF), healthy myotubes (HC-MT) and JDM myotubes (JDM-MT) were treated with Poly I:C to induce IFN beta, followed by the lead active siRNAs (4001 and 4002) or siRNAs with variable activity for comparison (4003, 4017 and 4030). RNA from these cultures was subjected to bulk RNA-sequencing.

Principal component analysis of the gene counts for all samples showed that the three replicates from each group clustered closely together (**Figure 4A**). The clearest difference in clustering between groups reflected the cell type (principal component 1 on the X-axis, **Figure 4A**), then with the untreated controls (UT) and Lipofectamine reagent only (UT-Lipo) groups generally distinct from the Poly I:C treated (PIC) groups (principal component 2 along the Y-axis, **Figure 4A**). From a heatmap of the top 100 most variably expressed genes across all samples (**Figure 4B**) hierarchical clustering was again driven by cell type, with myotubes (MT) versus dermal fibroblasts (DF) defined by 82 of these top 100 genes. In addition, consistent with the PCA analysis, the remaining 18 of these genes share a profile in UT/UT-Lipo treated cells distinct from Poly I:C treated samples, irrespective of cell type (**Figure 4B**). As this heatmap is derived only from the most variable genes, we would not expect the individual siRNA treatments to demonstrate their full differential activity; however, it is interesting that even the these 18 genes show evidence for the siRNA dosing reducing the sustained induction of IFN-stimulated transcripts upon Poly I:C induction, such as the 2’-5’-oligoadenylate synthetases (*OAS*)1, *OAS2* and *OASL* (**Figure 4C**). Here, lead siRNAs 4001 and 4002 (PIC-4001 and PIC-4002) appear to be influencing these genes more than 4003, 4007 and 4030; this phenomenon is investigated further below.

**Figure 4:**
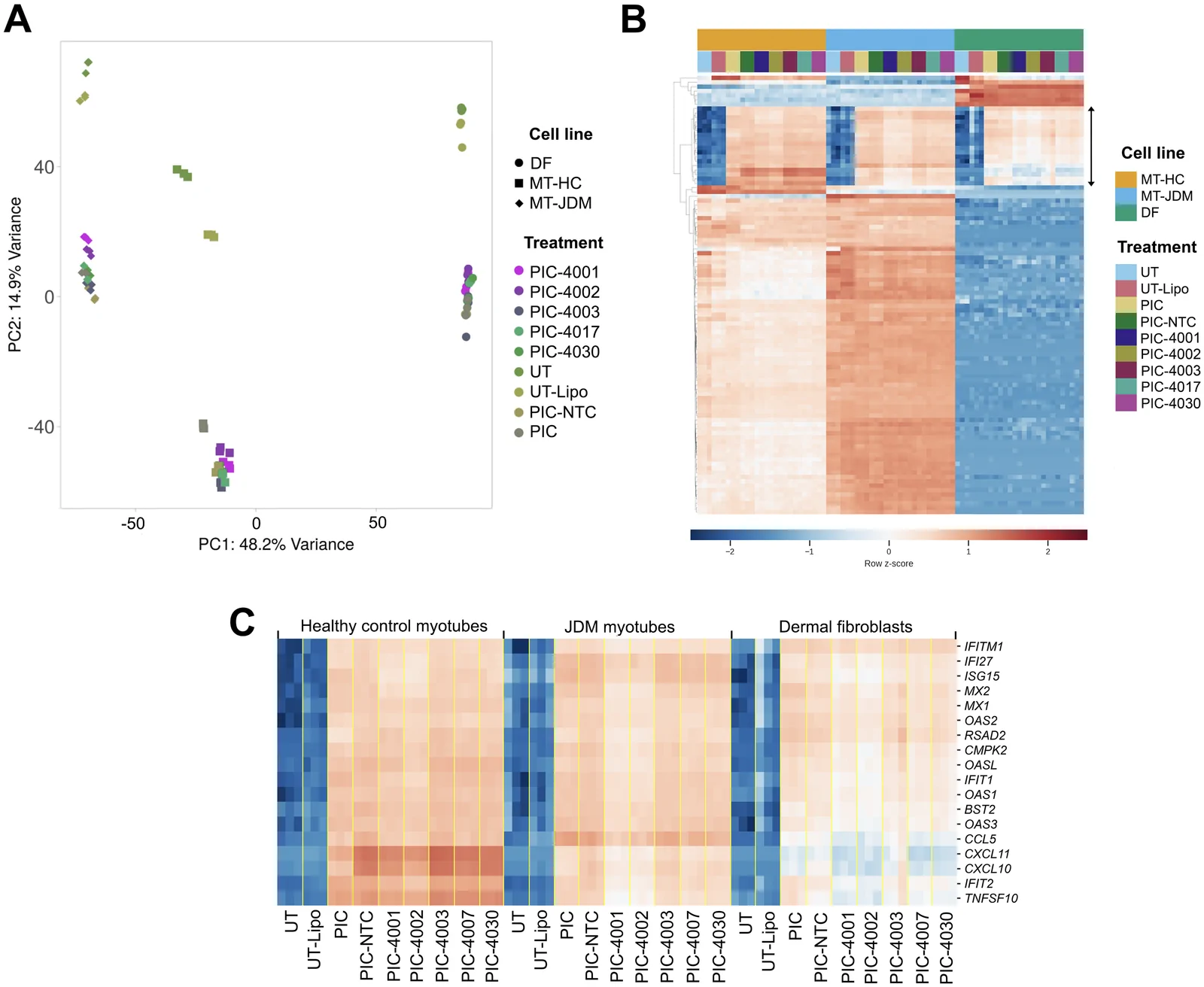
Sample clustering of the 100 most variable genes in bulk RNA sequencing for dermal fibroblasts and healthy control and juvenile dermatomyositis myotubes treated with Poly I:C followed by siRNAs targeting *IFNB1.* Cells were treated with 10 ng/ml Poly I:C for 24 hours followed by 20 nM (dermal fibroblasts, DF) or 40 nM (healthy control (HC-MT) or JDM (JDM-MT) myotubes) siRNA for 48 hours and RNA submitted for bulk RNA sequencing and analyses. Principal component analysis across cell lines and treatment groups **(A)**. Heatmap of hierarchical clustering generated with the 100 most variable genes using Pearson’s correlation coefficient for the distance matrix and average linkage. Shading indicates z-scores showing extent of increase (red) or decrease (blue) or no change (white) compared to mean expression per gene **(B)**. Clustering of 18 transcripts [designated by double-headed arrow in **(B)**] is re-shown with gene names labelled **(C)**. *DF, dermal fibroblasts; HC-MT, healthy control myotubes; JDM-MT, juvenile dermatomyositis myotubes; UT, non-treated; UT-Lipo, no treatment plus Lipofectamine 2000; PIC, poly I:C treatment; NTC, non-targeting control siRNA; 4001–4030, siRNAs*.

Considering pairwise comparisons between the groups, the data were analysed to generate metrics of the number of differentially expressed transcripts (FDR < 0.01, fold-change ≥ 2, **Figure 5A**) in addition to gene set enrichment analysis for the top 20 most significantly dysregulated KEGG pathways (FDR < 0.01, **Figure 5B**; **Supplementary Table S2**). As expected, Poly I:C treatment across all the cell lines tested resulted in significant differential expression of a large number of genes that corresponded to upregulation of viral, immune and autoimmune pathways (PIC versus UT, **Figures 5A, B**). A key control was also comparing Poly I:C treated cells with those also dosed with the non-targeting control siRNA (PIC-NTC). Across all three cell lines, the NTC siRNA appeared to significantly upregulate several common immune-related pathways, such as ‘IL-17 signaling’, ‘chemokine signaling’ and ‘cytokine/cytokine receptor interaction’ (**Supplementary Table S2**); these data demonstrate why it was important to use the NTC as a comparator for each *IFNB1* siRNA treatment as opposed to one of the non-siRNA-treated (UT or UT-Lipo) groups.

**Figure 5:**
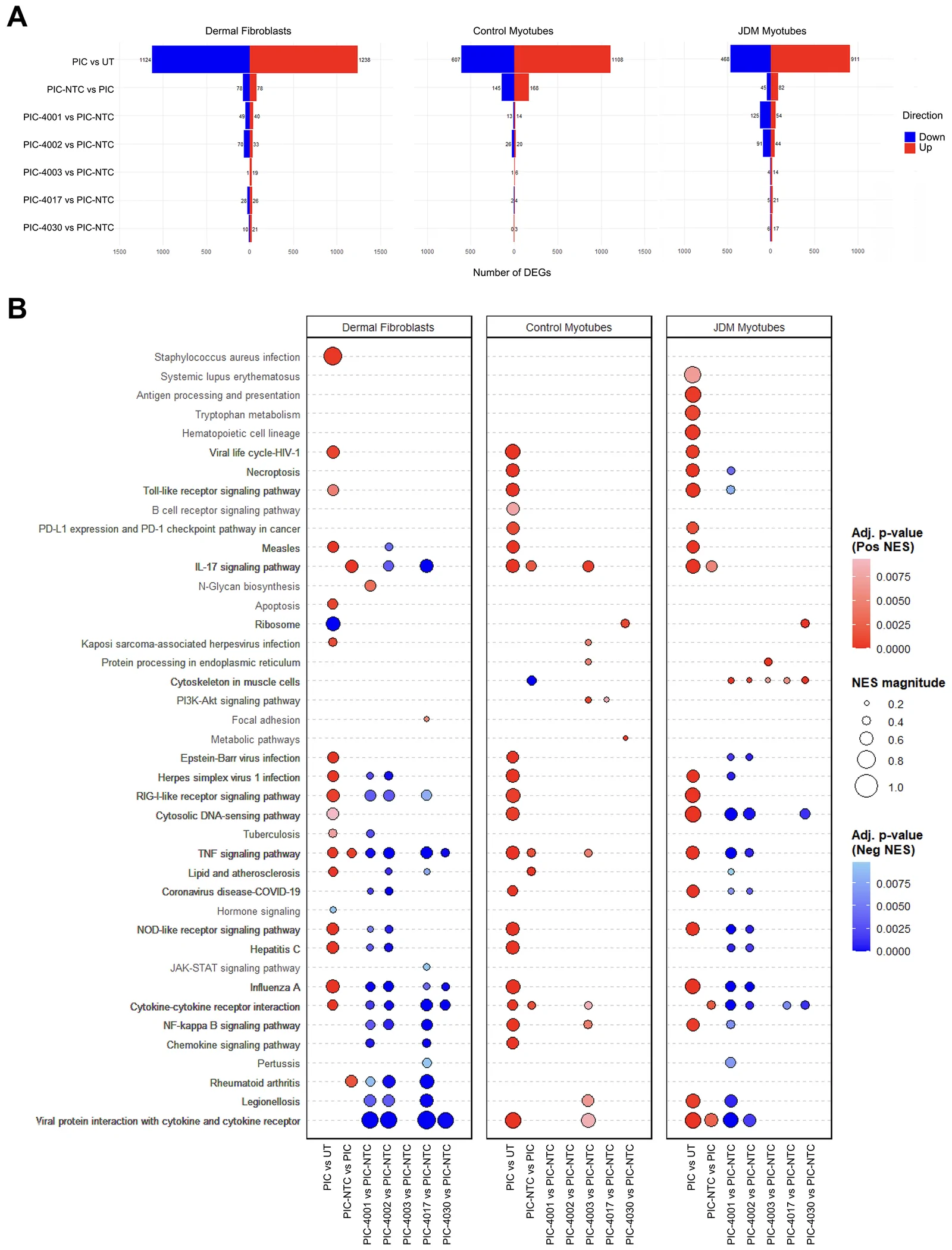
Gene expression and pathway analyses from bulk RNA sequencing for dermal fibroblasts and healthy control and juvenile dermatomyositis myotubes treated with Poly I:C followed by siRNA targeting *IFNB1.* Cells were treated with 10 ng/ml Poly I:C for 24 hours followed by 20 nM (dermal fibroblasts) or 40 nM (myotubes) siRNA for 48 hours and RNA submitted for bulk RNA sequencing and analysis. Differential gene expression analysis per cell line performed using DESeq2 with false discovery rate (FDR) adjusted p-value < 0.01 and fold change ≥ 2 and independent filtering of lower counts for selected comparisons. Red colour indicates increased and blue indicates decreased expression **(A)**. Pathway analysis performed using Gene Set Enrichment Analysis (GSEA) with pre-ranked fgsea and pathway significance cutoff of FDR < 0.01 for the top 20 most significantly dysregulated KEGG pathways. Shading indicates significance (adjusted p-value), size indicates normalised enrichment score, and colours indicate upregulation (red) or downregulation (blue) **(B)**. *UT, untreated; NTC, non-targeting control siRNA; 4001–4030, siRNAs.; PIC, poly I:C treatment; NES, normalised enrichment score*.

Pairwise comparisons between each *IFNB1* siRNA and the NTC were carried out from each cell line in order to provide evidence that individual siRNAs are modulating transcripts and pathways indicative of preventing or reversing interferon-associated activity. In comparisons of low activity siRNAs with the NTC (PIC-4003, -4017 and -4030 versus PIC-NTC), between zero and 28 significantly down- or up-regulated expressed genes were identified (**Figure 5A**), with few related to immune or viral infection pathways (**Figure 5B**). In contrast, the comparisons derived from the two lead siRNAs showed a greater number of differentially expressed genes, with more downregulated transcripts than upregulated, particularly in JDM myotubes (JDM-PIC-4001 and JDM-PIC-4002 versus JDM-PIC-NTC, **Figure 5A**). Interestingly, downregulated genes in dermal fibroblasts and JDM myotubes are represented in multiple infection and immune-associated pathways that are upregulated after Poly I:C treatment (**Figure 5B**). For example, the KEGG database shows that, similarly to the known mechanism of Poly I:C, the viral ‘influenza A’ response pathway includes TLR-mediated induction of IFN beta which feeds into the JAK-STAT signalling pathway which is known to be involved in DM/JDM pathology (26). In addition, the viral response can induce TNF alpha production, and the ‘TNF signalling pathway’ can in turn stimulate interferon regulatory factors that induce IFN beta (https://www.kegg.jp/entry/map04668) (27). Overall, the pathway regulation demonstrates the impact of reducing IFN beta expression with siRNAs to partially ‘rescue’ cells from the impact of Poly I:C treatment. Secondly, it is noteworthy that there are more differentially expressed genes upon *IFNB1* siRNA treatment in the JDM myoblasts versus the control myoblasts, suggesting the patient-derived cells are more sensitive to the siRNA treatment (**Figure 5B**).

To focus more specifically on IFN-associated ‘rescue’ by the lead siRNAs, the top 7000 most varied genes were clustered from JDM and control myotubes after siRNA 4001 or 4002 treatment, alongside the control untreated and Poly I:C treated samples. From these data, IFN-associated genes were selected using the Molecular Signatures Database (MSigDB) hallmark list (28) alongside core IFN pathway genes such as IFN ligands (IFNA/B/G/L subtypes), receptors (IFNAR1/2, IFNGR1/2, IFNLR1), and JAK/STAT/IRF signaling components. Visualizing the hierarchical clustering of these 190 genes shows the majority are highly induced by Poly I:C treatment, with many showing lower expression levels after siRNA dosing in JDM-derived versus control myotubes (**Figure 6A**). A 28-gene Interferon Response Gene Score (IRG-S) gene panel has been used clinically to provide the interferon signature in JDM patients (29). Combining the z-scores for 27 of the 28 genes that were present in the RNA-seq data results in a small but non-significant drop in their expression in JDM myotubes without active siRNA dosing (JDM-PIC/JDM-PIC-NTC versus controls HC-PIC/HC-PIC-NTC, **Figure 6B**). However, treatment with the siRNAs 4001 and 4002 blunts the combined IFN response gene expression significantly in JDM cells when compared to controls (HC-PIC-4001 versus JDM-PIC-4001; p < 0.01 and HC-PIC-4002 versus JDM-PIC-4002; p < 0.05, **Figure 6B**), further highlighting that core JDM disease-defining transcripts are modulated by the lead siRNAs. In summary, these data indicate that knockdown of *IFNB1* with siRNAs can dampen the immune response to Poly I:C treatment in JDM-derived cell lines.

**Figure 6:**
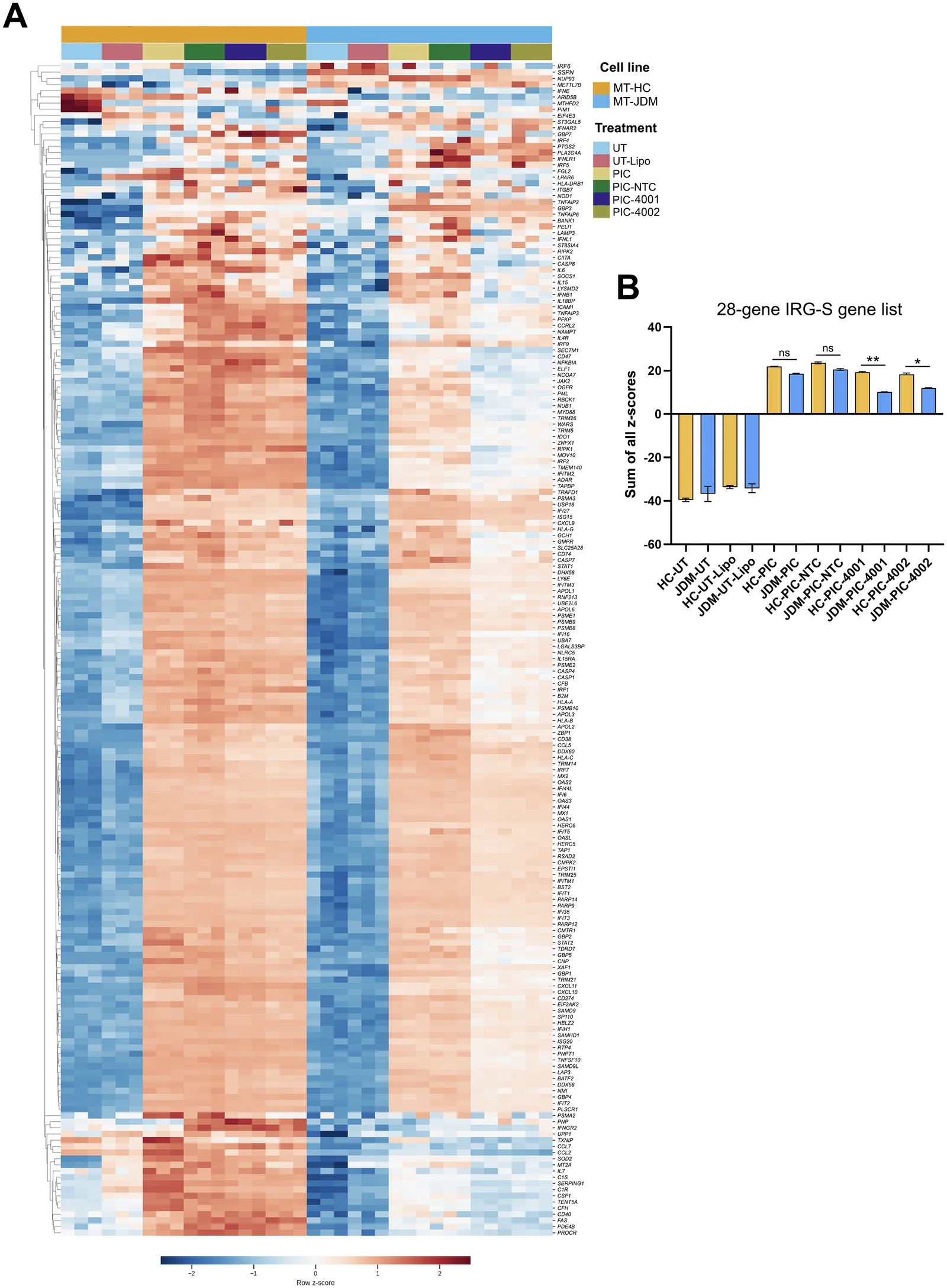
Sample clustering in bulk RNA sequencing for IFN-associated transcripts from healthy control and juvenile dermatomyositis myotubes treated with Poly I:C followed by siRNA targeting *IFNB1.* From the 7000 most variable genes in the RNA-seq dataset, a heatmap of hierarchical clustering for selection of 190 IFN-associated genes is shown using Pearson’s correlation coefficient for the distance matrix and average linkage **(A)**. Shading indicates z-scores showing extent of increase (red) or decrease (blue) or no change (white) compared to mean expression per gene. Cumulative z-scores for 28 selected JDM IFN signature genes from the same dataset **(B)**. Standard error of the mean is shown and data are compared between pairs of healthy control (HC) and JDM (JDM) myotubes for each treatment using one-way ANOVA with Turkey’s multiple comparisons test. * Indicates p-value <0.05; **<0.01; ns, non-significant; *C-MT, healthy control myotubes; JDM-MT, JDM myotubes; UT, untreated; UT-Lipo, untreated plus Lipofectamine 2000; PIC, poly I:C treatment; NTC, non-targeting control siRNA; 4001–4030 siRNAs*.

### 3.5 Extended siRNA library screening confirmed 4002 as lead siRNA for knockdown of *IFNB1* expression

The initial screen only included 27 siRNAs; therefore, to screen the *IFNB1* sequence in more depth, we synthesised a second panel of 47 siRNAs (**Supplementary Figure S1A**). To facilitate higher-throughput screening in a 96-well plate format, A549 lung carcinoma cells were used since DM/JDM can feature interstitial lung disease. A549 cells were treated with Poly I:C, followed by 50 nM of each siRNA by transfection, including siRNAs 4001, 4002 and 4003 from the initial screen for comparison. Treatment of A549 cells with 100 ng/ml Poly I:C induced a 560-fold increase in *IFNB1* expression compared to the no treatment group (Poly I:C versus NT in **Figure 7A**). In this screen, treatment with 17 siRNAs (including the positive controls 4002 and 4003) resulted in significant knockdown compared with the siRNA NTC (**Figure 7A**). The two siRNAs with greatest knockdown of *IFNB1* expression compared with the NTC, 4203 and 4002, were taken forward for further evaluation in skin and muscle cells. Notably, both of these siRNAs target untranslated regions, with 4002 located in the 5’ UTR and 4203 in the 3’UTR of *IFNB1* (**Supplementary Figure S1A**). Treatment with 10 ng/ml Poly I:C induced increased *IFNB1* expression by 1006-fold in dermal fibroblasts, 319-fold in control myotubes and 250-fold in JDM myotubes (Poly I:C + RNAiMAX versus NT in **Figures 7B–D**). Treatment with siRNA 4002 resulted in greater knockdown of *IFNB1* expression than 4203 in dermal fibroblasts and also control and JDM myotubes (**Figures 7B–D**), thus confirming siRNA 4002 as the lead siRNA candidate across relevant cell types.

**Figure 7:**
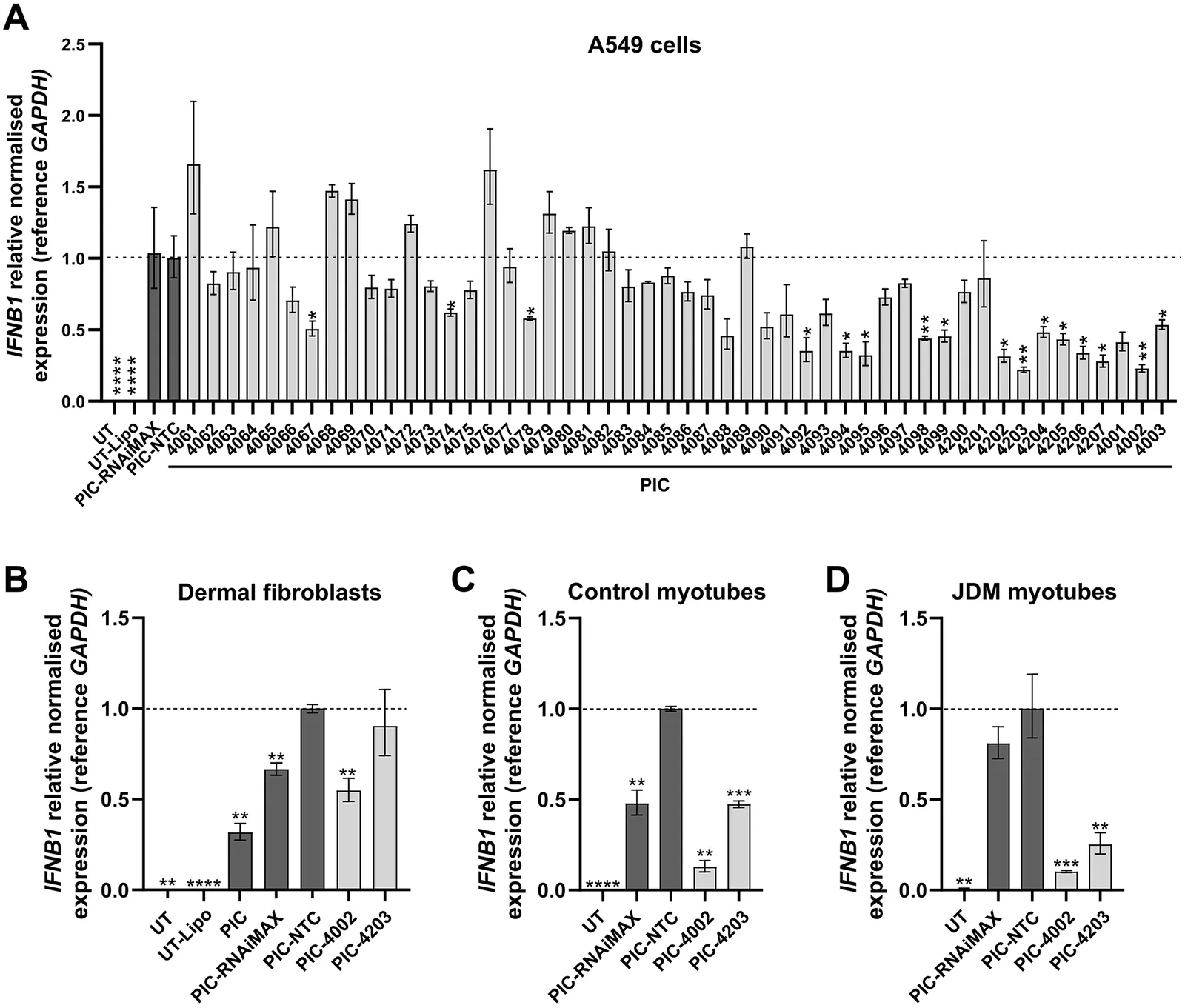
Expression of interferon beta in A549 lung cells, healthy control and juvenile dermatomyositis myotubes and dermal fibroblasts treated with Poly I:C followed by siRNA targeting *IFNB1*. A549 lung adenocarcinoma cells were transfected with 100 ng/ml Poly I:C for 24 hours followed by 50 nM siRNA for 48 hours. Normalised relative expression of *IFNB1* measured by qRT-PCR with reference gene *GAPDH* and control group non-targeting control siRNA (NTC) **(A)**. Immortalised dermal fibroblasts **(B)**, and healthy control **(C)** and juvenile dermatomyositis **(D)** patient-derived myotubes were transfected with 10 ng/ml Poly I:C followed by 50 nM siRNA. Normalised relative expression of *IFNB1* with reference gene *GAPDH* and control group non-targeting control siRNA (NTC). Normalised relative expression of *IFNB1*, standard error of the mean and p-values were calculated using Bio-Rad CFX Maestro software. * Indicates p-value <0.05; **<0.01, ***<0.001, ****<0.0001. *UT, untreated; UT-Lipo, no treatment plus Lipofectamine 2000; PIC, poly I:C treatment; PIC-RNAiMAX, poly I:C treatment plus Lipofectamine RNAiMAX after 24 hours; NTC, non-targeting control siRNA; 4001–4030 siRNAs*.

## 4 Discussion

Here, we have carried out a comprehensive screen for siRNAs that are active against the IFN beta transcript, *IFNB1*, in DM/JDM-relevant and JDM-derived cells and identified active compounds with favourable tolerability profiles. Multiple transcriptome and proteome studies from JDM-derived cells and muscle biopsies support the activity of type-I interferons and IFN-stimulated genes (ISGs) as being central to detrimental etiological pathways, including some correlation with ISG expression and disease activity (5, 30). Importantly, our data demonstrate that targeting *IFNB1* specifically can reverse viral, immune and autoimmune pathways in JDM-derived cells indicative of aberrant IFN beta activity, in addition to specific core IFN-stimulated transcripts that are used to characterise JDM. These findings suggest that *IFNB1* knockdown using siRNAs could be a promising therapeutic approach (**Figure 8**).

**Figure 8:**
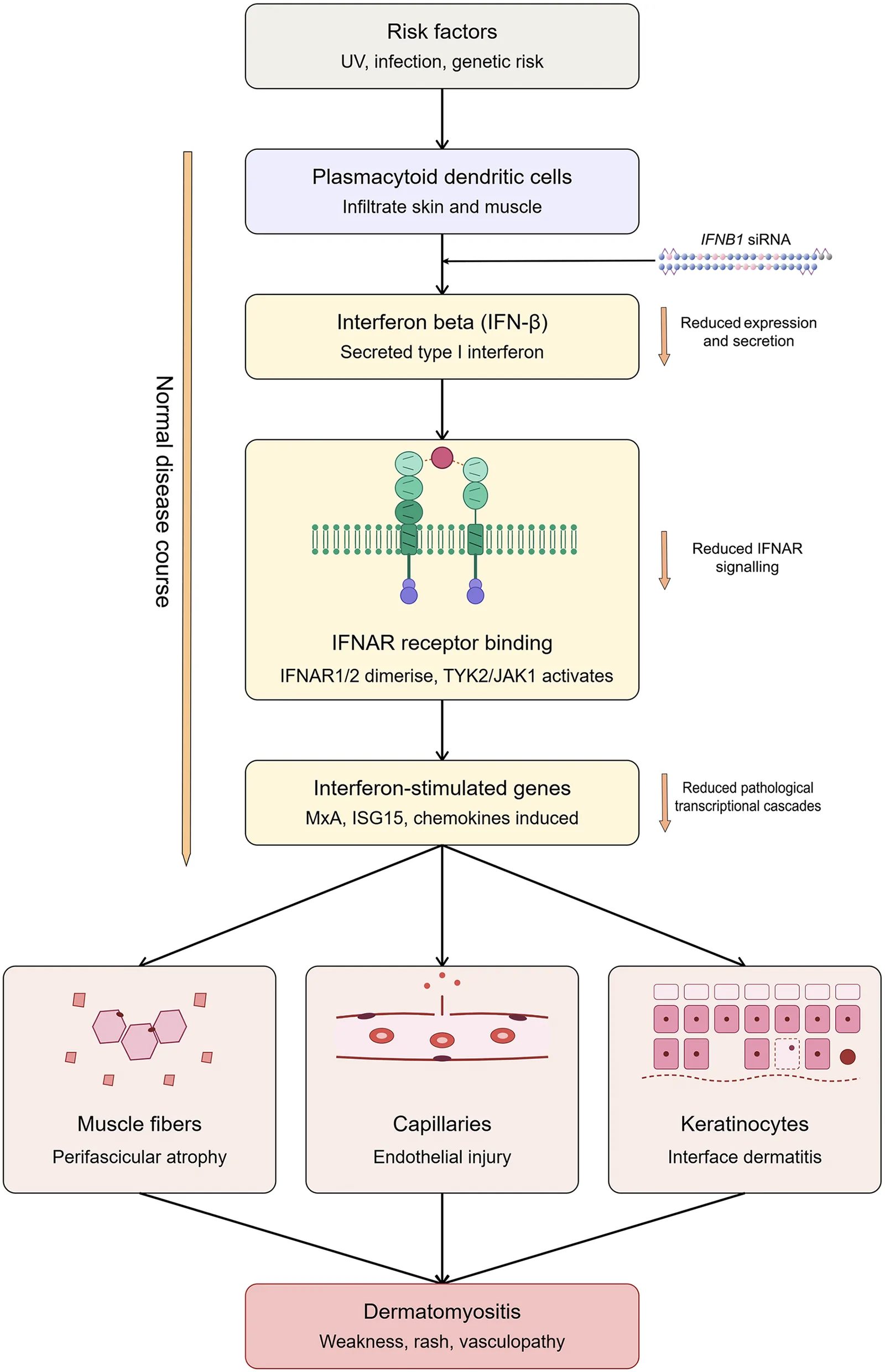
Proposed therapeutic mechanism of action using an *IFNB1* targeting siRNA.

One concern of the long-term suppression of IFN beta may be a depletion of anti-viral defense mechanisms and associated immune exhaustion pathways such as loss of T-cell effector functions (31, 32). However, the ultimate aim of the siRNA strategy here is to negate aberrant activity of type I IFN induction, whilst maintaining levels that are tolerated for normal immune system function. It must be considered, however, that antisense RNA therapeutics can generate their own immunostimulatory effects, such as those driven by unexpected sequence-driven off-target effects or the constituent nucleotides stimulating Toll-like receptors (TLRs) or pattern recognition receptors (33, 34). Indeed, this is exemplified by the induction of immune-related pathways in our RNA-seq analyses when dosing with the non-targeting control siRNA in Poly I:C treated cells versus Poly I:C alone (**Supplementary Table S2**); importantly, we utilised the NTC siRNA as the comparator in all the RNA-seq analyses to account for this variable. The chemical modification pattern used here is well-established, with strategic placement of 2’-OMe and 2’-F nucleobases to help mitigate immunogenicity while preserving gene-silencing activity (34, 35). The continued advancement of novel siRNA chemical modifications and their relative proportions in a given sequence design will undoubtedly improve both the efficacy and safety of future RNA therapies (36).

The validity of IFN beta as a therapeutic target his further supported by clincal data demonstrating the beneficial effects of targeting the pathways downstream of IFN beta (37). For example, the monoclonal antibody therapy anifrolumab binds and blocks the type I IFN receptor and Janus Kinase (JAK) inhibitors, prevent signalling downstream of type I IFN binding to the IFN alpha/beta receptor (56). However, such approaches block both IFN alpha and IFN beta activation of this pathway, resulting in increased vulnerability to infections (38). Furthermore, there are some concerns regarding other serious side effects of JAK inhibitors, including embolisms, thrombosis and neoplasms (38, 39). As an alternative strategy, a monoclonal antibody targeting IFN beta, dazukibart, has shown promise in patients with moderate to severe DM (NCT03181893), with significant improvements in skin scores and a trend toward improvement in muscle outcomes compared with placebo (40). Of note, in a case study of two JDM patients with severe refractory disease that did not respond to JAK inhibition, dazukibart treatment resulted in dramatic improvement in both muscle and skin measures (41). One potential advantage of a nucleic acid therapy approach is the specificity for the target transcript *IFNB1 -* thus not influencing IFN alpha genes - whilst also avoiding the accumulation of bound IFN beta protein. Yet the type I IFN signature in JDM may be derived from multiple cytokines (IFN alpha subtypes, IFN beta, IFN epsilon, IFN kappa, IFN omega) that converge on the same IFNAR1/2 receptor (5, 8). Knocking down *IFNB1* specifically may leave IFN alpha–driven signaling intact, limiting how much of the pathogenic signature is suppressed. Indeed, there are example of JDM IFN signatures that are predominantly IFN alpha; in a prospective study of 101 JDM patients, IFN beta was the most reliable biomarker of muscle disease activity measured by the Childhood-Myositis Assessment Scale (CMAS) across all subgroups, although melanoma differentiation-associated gene 5 (MDA5) autoantibody positive patients also had elevated IFN alpha which was associated with CMAS score (42). Therefore, there are some patients where a downstream JAK inhibitor blocking the shared receptor pathway could have therapeutic advantages over an siRNA. Of note, targeting IFN alpha using an siRNA is more complex than IFN beta due to the large number of non-redundant *IFNA1* isoforms clustered in the human genome (43–45).

Fundamental questions around development of an *IFNB1* siRNA for DM/JDM include compound delivery, and whether systemic dosing will be sufficient or whether targeted delivery to muscle, skin, lung or even specific cells of the immune system will be necessary for clinical efficacy. Recent advances in siRNA conjugation strategies include antibodies or peptides that bind the transferrin receptor 1 for delivery to muscle (46, 47), and an albumin binding conjugation to target inflamed joints has been shown to be promising in *in vivo* models of osteoarthritis (48). For the skin, direct topical application could be considered; for example, transdermal delivery of an siRNA using a composite ionic liquid showed improvement in skin of a psoriasis mouse model (49), while JAK1-targeting siRNAs conjugated to the lipophilic docosanoic acid show promise for dampening IFNγ-associated cytokine activity in a porcine *in vivo* model (50). Targeting immune cells may also be essential, in particular given their fundamental role in propagating IFN signaling (51). Plasmacytoid dendritic cells (pDCs) are the key driver for IFN release and perivascular infiltration, therefore targeting these cells would likely be more beneficial than macrophages, neutrophils or cells of the adaptive immune system (52). Specifically targeting these cells would be a challenging given their scarcity in the circulation (approximately 0.5% of peripheral blood mononuclear cells (PBMCs)) as well as their accessibility in tissue post-infiltration (53); however, future strategies could include antibody-siRNA conjugates or nanoparticles utilising known pDC markers, such as BDCA-2, BDCA-4 or ILT7, for example (21, 54). Whether such delivery systems will be sufficient to target the specific cell types driving ISG cascades in DM/JDM remains to be tested. Furthermore, the long-acting pharmacokinetics of N-acetylgalactosamine (GalNAc)-conjugated siRNAs may not be recapitulated with different targeting moieties (36).

In conclusion, we have identified a lead siRNA candidate with effective IFN beta transcript and protein knockdown, low cytotoxicity and specificity of knockdown for pathways downstream of *IFNB1* in muscle and skin *in vitro.* Our study is limited to a single JDM patient-derived cell line; expanding compound testing to cells from multiple patients with differing clinical presentations will be important to ascertain whether the siRNAs are equally as effective given the natural disease heterogeneity (2). Further work will also be required to evaluate whether knockdown of IFN beta in this manner produces functional benefit in mouse models of myositis, and in particular versus IFN antibody strategies. Importantly, a nucleic acid therapy that can knockdown IFN beta expression may have applications beyond DM/JDM including other autoimmune conditions demonstrating IFN overexpression, type I interferonopathies or as co-treatment with other nucleic acid therapies that induce an unwanted inflammatory response (55).

## Data Availability

The data presented in the study are deposited in the GEO database repository, accession number GSE322641.

## Supplementary material

Supplementary Figure 1 Binding positions of siRNA along the *IFNB1* mRNA transcript sequence and pattern of chemical modifications. Oligonucleotides were designed targeting the *IFNB1* mRNA transcript NM_002176.4. A library of 21-nt sequences was generated across the entire transcript and then filtered using exclusion criteria. The 27 siRNAs in the first round of synthesis are shaded blue and the 47 siRNA in the second round of synthesis are shaded green **(A)**. Figure generated using SnapGene software. The chemical modification pattern of the siRNAs included 2′-O-Methyl and 2′-fluoro modified nucleotides with 2-nt dT overhang at the 3’ end of the antisense strand and two phosphorothioate bonds at both ends of the duplex **(B)**. 5’UTR 5’ untranslated region, CDS coding sequence, 3’UTR 3’ untranslated region, 2’-OMe 2′-O-Methyl nucleotide, 2’F 2′-fluoro modified nucleotide, PO phosphodiester, PS phosphorothioate

Supplementary Table 1 Differences in apoptosis and necrosis response following 24 hours of treatment with siRNAs targeting *IFNB1* compared to non-targeting control siRNAs. Immortalised myoblasts, differentiated myotubes, dermal fibroblasts and healthy and JDM patient-derived myotubes were transfected with 0.1–500 or 1000nM siRNA immediately followed by addition of RealTime-Glo Annexin V Apoptosis and Necrosis Assay reagents in triplicate for each siRNA. The difference in net luminescence (apoptosis) and fluorescence (necrosis) signals from cells treated with each siRNA compared to the non-targeting control (NTC) siRNA at each concentration was analysed using a two-way ANOVA with Dunnett’s multiple comparisons test on GraphPad Prism version 10.5.0. Yellow highlighting indicates where the signal with treatment with NTC is significantly lower than the siRNA and green indicates where the NTC is significantly higher than the siRNA (adjusted p-value<0.01). NTC non-targeting control siRNA.

Supplementary Table 2 Top 20 most significantly dysregulated KEGG pathways in dermal fibroblasts, healthy control and JDM myotubes. Cells were treated with 10 ng/ml Poly I:C for 24 hours followed by 20 nM (dermal fibroblasts) or 40 nM (myotubes) siRNA for 48 hours and RNA submitted for bulk RNA sequencing and analysis. Pathway analysis performed using Gene Set Enrichment Analysis (GSEA) with pre-ranked fgsea and pathway significance cutoff of FDR < 0.01 for the top 20 most significantly dysregulated KEGG pathways. *UT, no treatment; NTC, nontargeting control siRNA; NES, normalised enrichment score.*
